# A nomogram for evaluation and analysis of difficulty in retroperitoneal laparoscopic adrenalectomy: A single-center study with prospective validation using LASSO-logistic regression

**DOI:** 10.3389/fendo.2022.1004112

**Published:** 2022-11-25

**Authors:** Shiwei Sun, Jinyao Wang, Bin Yang, Yue Wang, Wei Yao, Peng Yue, Xiangnan Niu, Anhao Feng, Lele Zhang, Liang Yan, Wei Cheng, Yangang Zhang

**Affiliations:** ^1^ Third Hospital of Shanxi Medical University, Shanxi Bethune Hospital, Shanxi Academy of Medical Sciences, Tongji Shanxi Hospital, Taiyuan, China; ^2^ Shanxi Bethune Hospital, Shanxi Academy of Medical Sciences, Tongji Shanxi Hospital, Third Hospital of Shanxi Medical University, Taiyuan, China; ^3^ Tongji Hospital, Tongji Medical College, Huazhong University of Science and Technology, Wuhan, China; ^4^ Department of Urology, Tangdu Hospital, Air Force Medical University, Xi’an, China

**Keywords:** adrenalectomy, laparoscopy, retroperitoneal space, LASSO, nomogram

## Abstract

**Background:**

While it is known that inaccurate evaluation for retroperitoneal laparoscopic adrenalectomy (RPLA) can affect the surgical results of patients, no stable and effective prediction model for the procedure exists. In this study, we aimed to develop a computed tomography (CT) -based radiological-clinical prediction model for evaluating the surgical difficulty of RPLA.

**Method:**

Data from 398 patients with adrenal tumors treated by RPLA in a single center from August 2014 to December 2020 were retrospectively analyzed and divided into sets. The influencing factors were selected by least absolute shrinkage and selection operator regression model (LASSO). Additionally, the nomogram was constructed. A receiver operating characteristic curve was used to analyze the prediction efficiency of the nomogram. The C-index and bootstrap self-sampling methods were used to verify the discrimination and consistency of the nomogram.

**Result:**

The following 11 independent influencing factors were selected by LASSO: body mass index, diabetes mellitus, scoliosis, hyperlipidemia, history of operation, tumor diameter, distance from adrenal tumor to upper pole of kidney, retro renal fat area, hyperaldosteronism, pheochromocytoma and paraganglioma, and myelolipoma. The area under the curve (AUC) of the training set was 0.787, and 0.844 in the internal validation set. Decision curve analyses indicated the model to be useful. An additional 117 patients were recruited for prospective validation, and AUC was 0.848.

**Conclusion:**

This study developed a radiological-clinical prediction model proposed for predicting the difficulty of RPLA procedures. This model was suitable, accessible, and helpful for individualized surgical preparation and reduced operational risk. Thus, this model could contribute to more patients’ benefit in circumventing surgical difficulties because of accurate predictive abilities.

## Introduction

Adrenal tumors are common among urological conditions, with a median incidence rate of approximately 3.0% (1.05%–8.7%) (1). Involved pathologies include nonfunctional adrenal tumors, primary aldosteronism (PA), Cushing’s syndrome, pheochromocytoma and paraganglioma (PPGL), myelolipomas, ganglioneuromas, adrenocortical carcinomas, and adrenal metastasis (2, 3). Computed tomography (CT) is among the preferred localization and diagnostic methods, as it can typically detect adrenal tumors with a diameter of >5 mm (4). In addition, the pathology of lesions can be evaluated preliminarily by CT (5).

As the gold standard treatment for adrenal tumors, laparoscopic surgery has the advantages of accelerating postoperative recovery and shortening postoperative hospital stay (POHS) as compared with open surgery (6–8). Laparoscopic surgery is further divided into transperitoneal laparoscopic adrenalectomy (TPLA) and retroperitoneal laparoscopic adrenalectomy (RPLA), with TLPA first competed in 1992 by Gagner et al. (9) and Higashihara et al. (10) and RPLA first proposed by Gaur et al. (11), later refined by Walz et al. (12). Compared with TLPA, RPLA has less postoperative pain, fewer complications, and lower incidence of intraoperative adverse events such as hemodynamic instability and massive bleeding (13, 14). However, there is currently no effective evaluation system for the difficulties of RPLA. Therefore, exploration of new strategies to improve evaluation efficiency and optimize treatment of adrenal tumors is essential.

Machine learning is a new technique used widely in medical research in recent years. Aside from processing a large amount of data by identifying key factors, it can also capture the relationship between nonlinear variables and accurately predict clinical outcomes. As listed above, this is an indispensable approach to solving complex problems in various fields (15). For instance, current research explored the differences between adrenal pheochromocytoma and lipid-poor adenoma from machine learning (16).

LASSO was established by Tibshirani and found to be one of the most effective classifier construction algorithms to predict clinical outcomes in various classification or regression studies (17). Furthermore, a study used LASSO regression on the preoperative diagnosis of pheochromocytoma, and the model set yielded an AUC of 0.893 (18).

This study used LASSO to retrospectively analyze the influencing factors correlating to the difficulties of RPLA by developing a nomograph. Additionally, this study focused on improving preoperative preparations, reducing the operational risks and improving patients’ clinical outcomes.

## Methods

### Patients and selection criteria

**Figure 1:**
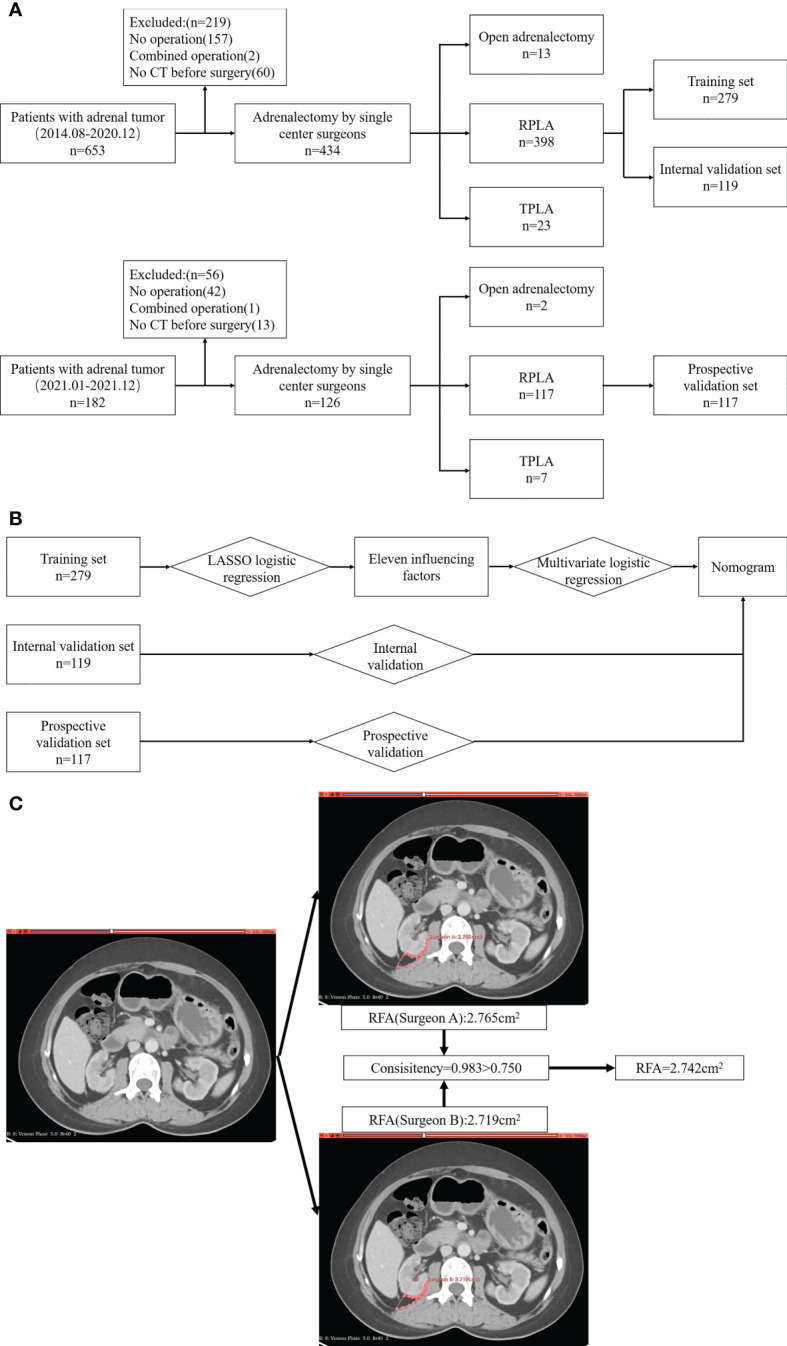
Study flowchart. **(A)** Inclusion and exclusion process; **(B)** Analysis and verification process; **(C)** Drawing of ROI and calculation of RFA.

To meet our aim of using LASSO to retrospectively analyze factors influencing the difficulty of RPLA in patients in a single hospital and developing a nomograph to individualize preoperative preparation and reduce operational risk, we retrospectively collected the data of adrenal tumor patients in Shanxi Bethune hospital from August 2014 to December 2020. The models were established based on the retrospective patient data, while adrenal tumor patients from January 2021 to December 2021 were prospectively collected for prospective validation. Inclusion criteria included: 1) confirmation of adrenal tumors by abdominal CT examination 1–15 days before operation, 2) routine laboratory examinations (serum aldosterone [supine and erect position], direct renin concentration [supine and erect position], serum cortisol [8:00, 16:00, and 24:00], plasma catecholamine, urine catecholamine, and vanillylmandelic acid) to determine the hormone activity of adrenal tumors performed before operation, and 3) treated by RPLA. Exclusion criteria included: 1) no surgery conducted, 2) multiple operations undergone concurrently, and 3) incomplete CT data. A total of 398 patients were included in the retrospective study, with an additional 117 patients recruited for prospective validation (**Figure 1A**).

### Procedures

As referenced from the previous studies (13, 14, 19–21), patients who meet any of the following conditions are considered to be difficult to operate on: 1) operation time ≥ P_75_ (150_ min_), 2) blood transfusion required during operation due to injury of surrounding organs or blood vessels, 3) convert to open surgery, 4) postoperative complications with Clavien–Dindo grade ≥ 3, 5) POHS ≥ P_95_ (15 d). Otherwise, patients are considered to be easy to operate on.

For this study, patients were divided randomly into training and internal validation sets, with a proportion of 7:3. The data from the training set was used for influencing factor selection and model construction, whereas the data of the internal validation set was used to validate the model (**Figure 1B**).

Radiological features were obtained using Siemens SOMATOM definition flash or force dual-source CT, with Siemens SOMATOM Definition AS Sliver or GEOptima 660 spiral CT scanning (Siemens, Munich, Germany). CT scanning was performed with 1.0 mm slice thickness and 0.7 mm intervals. The method for measurement of features was as follows. For tumor diameter (TD), we took the larger value of the longest diameter in the largest section of the tumor and the difference between the upper and lower poles of the tumor. For distance from adrenal tumor to upper pole of kidney (DAK), we took the vertical height difference between the lower pole of adrenal tumors and the upper pole of the ipsilateral kidney. For the distance from adrenal tumor to renal pedicle (DARP), we took the vertical height difference between the lower pole of the adrenal tumor and the plane of the ipsilateral renal vein. For the distance from great vessel to the adrenal tumor, we selected the slice closest to the tumor and great vessel (left: abdominal aorta; right: inferior vena cava), and measured the shortest distance between them. For the retrorenal fat area (RFA), we used 3D slicer software to draw the region of interest (ROI) for measurement. The ROI was drawn at the slice of the renal vein in the horizontal view, encompassed by the extended line of the renal vein and its perpendicular line, as well as the visceral tectorial membrane and parietal peritoneum. The above features were measured by two doctors, respectively. If the consistency of the two measurements was >0.75, we took the average of the two values; otherwise, the measurement was re-taken by another senior doctor (**Figure 1C**).

### Statistical analysis

R 4.1.4 (Vienna statistical computing foundation, Austria) and the glmnet (22) package were used to process data. As all continuous variables did not conform to the normal distribution, they are represented by the median [interquartile range]; categorical variables are expressed in frequency and percentage (%). The patients were randomly divided into the training and internal validation sets according to a ratio of 7:3, and the baseline characteristics between each set were compared by analysis of variance. Using the glmnet package, the univariate logistic regression and lasso logistic regression analysis were carried out to verify the independent influencing factors. The nomogram model was then established according to multivariate logistic regression, and the ROC curve and the AUC were used to verify the prediction efficiency of the model. The bootstrap resampling method and calibration curve were used to evaluate the consistency of the model, and the net benefit of patients was evaluated by clinical decision curve analysis (DCA). The prediction model was then verified again in the prospective group. When P-value <0.05, the difference was considered to be statistically significant.

## Results

### Baseline characteristics

This study involved 398 patients, with all baseline characteristics listed in **Table 1**. The median patient age is 50.50 [40.00, 58.00] years with 179 male (45.0%) and 219 female (55.0%). A total of 132 patients were considered to be challenging to operate on, with 105 having had an operation time ≥ P_75_, 13 having required blood transfusion due to intraoperative damage to surrounding organs or vessels, seven having undergone conversion to open surgery, 27 having had complications with a Clavien–Dindo grade ≥ 3 (**Table 2**), and 19 having had a postoperative hospital stay of ≥ P_95_. It was discovered by ANOVA that there was no significant difference in preoperative baseline characteristics among the sets.

**Table 1:** Baseline characteristics of the patients.

	Training set (n = 279)	Internal validation set (n = 119)	Prospective validation set (n = 117)	F	P
Gender				1.214	0.298
Male	130 (46.6)	49 (41.2)	60 (51.3)		
Female	149 (53.4)	70 (58.8)	57 (48.7)		
Age (yr)	51.00[42.00,58.50]	50.00[38.50,58.00]	51.00[39.00,58.00]	0.400	0.671
BMI (kg·m^-2^)	24.61[22.84,27.35]	25.35[22.88,27.91]	25.95[23.44,28.32]	2.912	0.055
Pathology				1.536	0.216
NFAT	115 (41.2)	45 (37.8)	38 (32.5)		
PA	81 (29.0)	31 (26.1)	43 (36.8)		
Cushing’s syndrome	29 (10.4)	12 (10.1)	17 (14.5)		
PPGL	21 (7.5)	12 (10.1)	7 (6.0)		
Myelolipoma	11 (3.9)	4 (3.4)	4 (3.4)		
Cyst	10 (3.6)	6 (5.0)	3 (2.6)		
Malignant tumor	4 (1.4)	2 (1.7)	2 (1.7)		
Ganglioneuroma	3 (1.1)	1 (0.8)	2 (1.7)		
Others	5 (1.8)	6 (5.0)	1 (0.9)		
Side				0.352	0.703
Left	166 (59.5)	72 (60.5)	65 (55.6)		
Right	113 (40.5)	47 (39.5)	52 (44.4)		
Hypertension				0.369	0.692
No	60 (21.5)	26 (21.8)	21 (17.9)		
Yes	219 (78.5)	93 (78.2)	96 (82.1)		
Diabetes mellitus				1.335	0.264
No	230 (82.4)	92 (77.3)	89 (76.1)		
Yes	49 (17.6)	27 (22.7)	28 (23.9)		
Scoliosis				1.351	0.26
No	267 (95.7)	112 (94.1)	115 (98.3)		
Yes	12 (4.3)	7 (5.9)	2 (1.7)		
Coronary disease				1.957	0.142
No	253 (90.7)	105 (88.2)	98 (83.8)		
Yes	26 (9.3)	14 (11.8)	19 (16.2)		
Cerebral infarction				0.082	0.921
No	253 (90.7)	107 (89.9)	107 (91.5)		
Yes	26 (9.3)	12 (10.1)	10 (8.5)		
Hyperlipidemia				0.184	0.832
No	249 (89.2)	106 (89.1)	102 (87.2)		
Yes	30 (10.8)	13 (10.9)	15 (12.8)		
History of malignancy				2.024	0.133
No	268 (96.1)	117 (98.3)	109 (93.2)		
Yes	11 (3.9)	2 (1.7)	8 (6.8)		
History of operation				0.603	0.547
No	198 (71.0)	89 (74.8)	80 (68.4)		
Yes	81 (29.0)	30 (25.2)	37 (31.6)		
Hb (g·L^-1^)	137.00[127.00,146.00]	135.00[126.00,146.00]	137.00[127.00,146.00]	0.318	0.728
TD (mm)	20.80[15.10,30.40]	22.00[15.50,35.20]	20.00[14.00,28.00]	2.621	0.074
DAK (mm)	-10.00[-20.80,-2.70]	-12.80[-21.20,-5.20]	-9.60[-17.60,-3.50]	1.642	0.195
DARP (mm)	28.80[19.20,37.90]	28.00[18.10,36.80]	28.70[18.40,40.00]	0.587	0.556
DGV (mm)	8.00[3.50,14.00]	6.00[3.00,12.00]	7.00[4.00,12.00]	0.628	0.534
RFA (mm^2^)	399.00[203.40,668.50]	429.30[222.00,703.55]	370.30[237.30,635.80]	0.627	0.535
Resection range				0.152	0.859
Partial	251 (90.0)	109 (91.6)	105 (89.7)		
Radical	28 (10.0)	10 (8.4)	12 (10.3)		
Operation time (min)	110.00[85.00,150.00]	120.00[95.00,150.00]	98.00[76.00,130.00]	5.015	0.007
Blood loss (ml)	20.00[0.00,50.00]	20.00[10.00,50.00]	15.00[0.00,25.00]	0.763	0.467
POHS (d)	7.00[6.00,9.00]	8.00[7.00,9.50]	6.00[2.00,7.00]	15.683	<0.001

BMI, body mass index; NFAT, non-function adrenal tumor; PA, primary aldosteronism; PPGL, pheochromocytoma and paraganglioma; TD, tumor diameter; DAK, distance from adrenal tumor to upper pole of kidney; DARP, distance from adrenal tumor to renal pedicle; DGV, distance from great vessel to adrenal tumor; RFA, retrorenal fat area.

Others (pathology) include eosinophil tumor, teratoma, schwannoma, hematoma, tuberculoma, foreign body granuloma, retroperitoneal bronchial cyst, hemangioma.

**Table 2:** Classification of complications.

Complications	Ⅰ	II	IIIa	IIIb	IVa	Summation
Fever	11					11
Hypokalemia	6					6
Hypofunction of cortex	5					5
Hyperkalemia	1					1
Mumps	1					1
Wound infection	1					1
Delayed bleeding	1	1				2
Incomplete ileus		1				1
Deep venous thrombosis		3	1			4
Foreign body granuloma				1		1
Systemic inflammatory response syndrome					1	1
Disturbances of vital signs (requiring ICU management)					24	24
Total	26	5	1	1	25	58

### Univariate logistics regression

**Figure 2:**
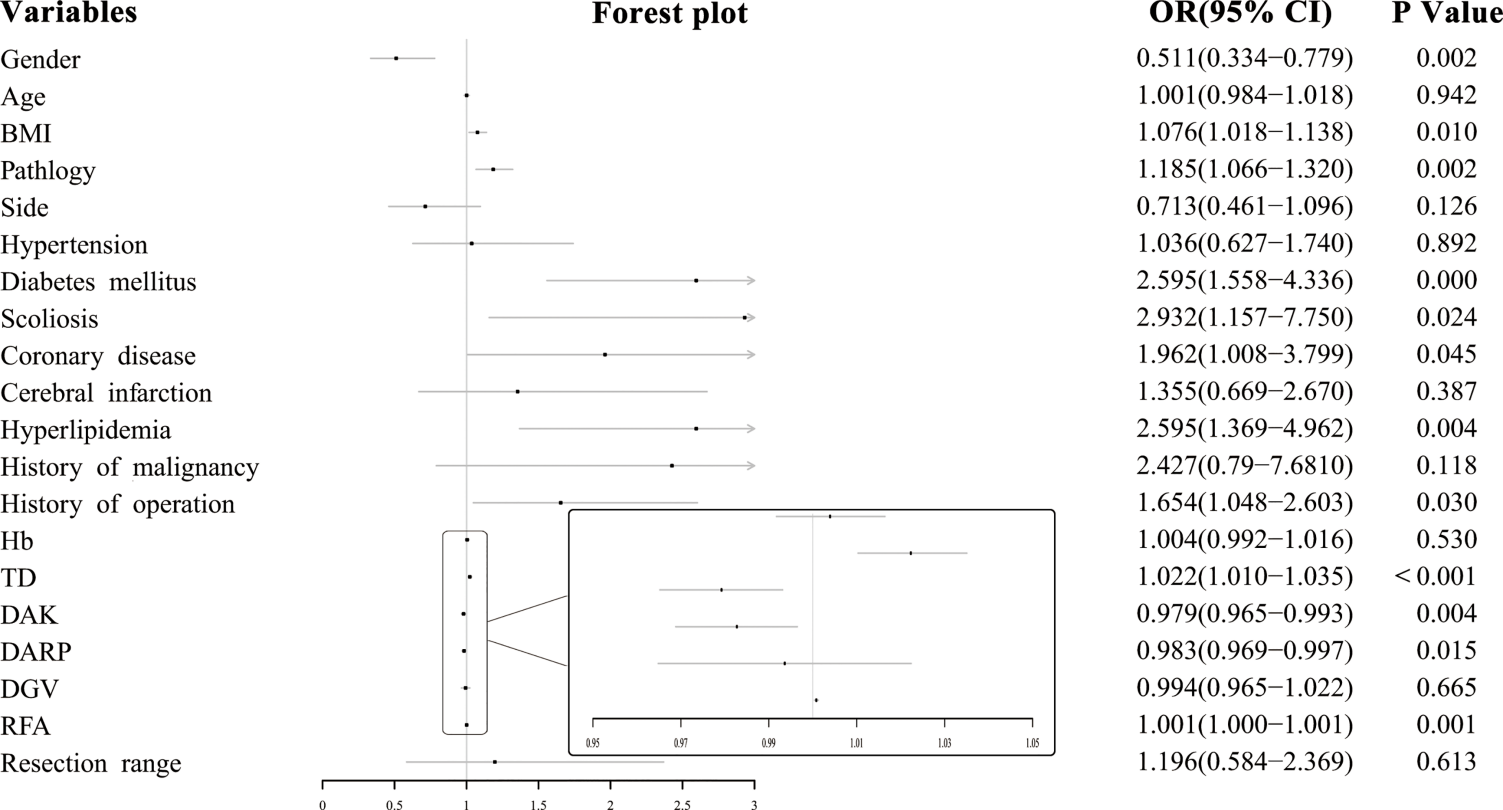
Forest plot of influencing factors of surgical difficulty.

Univariate logistics regression suggested that there were several influencing factors associated with the difficulty of RPLA, including gender (Odds ratio[OR]: 0.511, 95% Confidence interval[CI]: 0.334–0.779, P=0.002), body mass index (BMI) (OR: 1.076, 95% CI: 1.018–1.138, P=0.01), pathology (OR: 1.185, 95% CI: 1.066–1.320, P=0.002), diabetes mellitus (OR: 2.595, 95% CI: 1.558–4.336, P=0), scoliosis (OR: 2.932, 95% CI: 1.157–7.750, P=0.024), coronary disease (OR: 1.962, 95% CI: 1.008–3.799, P=0.045), hyperlipidemia (OR: 2.595, 95% CI: 1.369–4.962, P=0.004), history of operation (OR: 1.654, 95% CI: 1.048–2.603, P=0.03), TD (OR: 1.022, 95% CI: 1.010–1.035, P=0.001), DAK (OR: 0.979, 95% CI: 0.965–0.993, P=0.004), DARP (OR: 0.983, 95% CI: 0.969–0.997, P=0.015), and RFA (OR: 1.001, 95% CI: 1.000–1.001, P=0.001) (**Figure 2**).

### Variable selection

**Figure 3:**
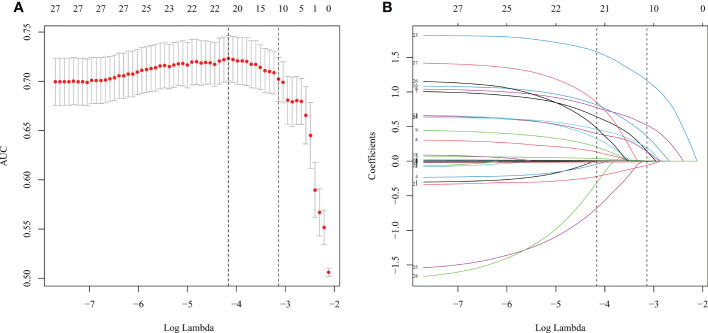
LASSO-Logistic regression. **(A)** The cross-validation results. **(B)** LASSO coefficient profiles of the 28 variables.

We converted multi-categorical variables into binary-categorical variables through dummy variables; final variable assignments are shown in **Table 3**. Generalized cross-validation was carried out for all variables through LASSO logistics regression, with the log (lambda) value of the harmonic parameter. The AUC of the model changed along with the change of the lambda. The corresponding number of variables filtered by the model is shown in **Figure 3A**. We constructed an influencing factor classifier by using the lasso logistic regression model (**Figure 3B**). After lasso logistic analysis, eleven influencing factors were selected including BMI, diabetes mellitus, scoliosis, hyperlipidemia, history of operation, TD, DAK, RFA, PA, PPGL, and myelolipoma (**Table 4**).

**Table 3:** Variable assignments.

Variable	Risk Factors	Assignments
X_1_	Gender	Male=0, female=1
X_2_	Age	Continuous variable
X_3_	BMI	Continuous variable
X_4_	Side	Left=0, right=1
X_5_	Hypertension	No=0, Yes=1
X_6_	Diabetes mellitus	No=0, Yes=1
X_7_	Scoliosis	No=0, Yes=1
X_8_	Coronary disease	No=0, Yes=1
X_9_	Cerebral infarction	No=0, Yes=1
X_10_	Hyperlipidemia	No=0, Yes=1
X_11_	History of malignancy	No=0, Yes=1
X_12_	History of operation	No=0, Yes=1
X_13_	Hb	Continuous variable
X_14_	TD	Continuous variable
X_15_	DAK	Continuous variable
X_16_	DARP	Continuous variable
X_17_	DGV	Continuous variable
X_18_	RFA	Continuous variable
X_19_	Resection range	Partial=0, Radical=1
X_20_	NFAT	No=0, Yes=1
X_21_	PA	No=0, Yes=1
X_22_	Cushing syndrome	No=0, Yes=1
X_23_	PPGL	No=0, Yes=1
X_24_	Myelolipoma	No=0, Yes=1
X_25_	Cyst	No=0, Yes=1
X_26_	Malignant tumor	No=0, Yes=1
X_27_	Ganglioneuroma	No=0, Yes=1
X_28_	Other pathological types	No=0, Yes=1

**Table 4:** Risk factors selected by LASSO-logistic regression model.

Variable	Risk Factors	Coefficient
X_3_	BMI	0.0033
X_6_	Diabetes mellitus	0.1469
X_7_	Scoliosis	0.5200
X_10_	Hyperlipidemia	0.3623
X_12_	History of operation	0.1411
X_14_	TD	0.0025
X_15_	DAK	-0.0078
X_18_	RFA	0.0004
X_21_	PA	-0.0605
X_23_	PPGL	1.1655
X_24_	Myelolipoma	0.1949

### Nomogram

**Figure 4:**
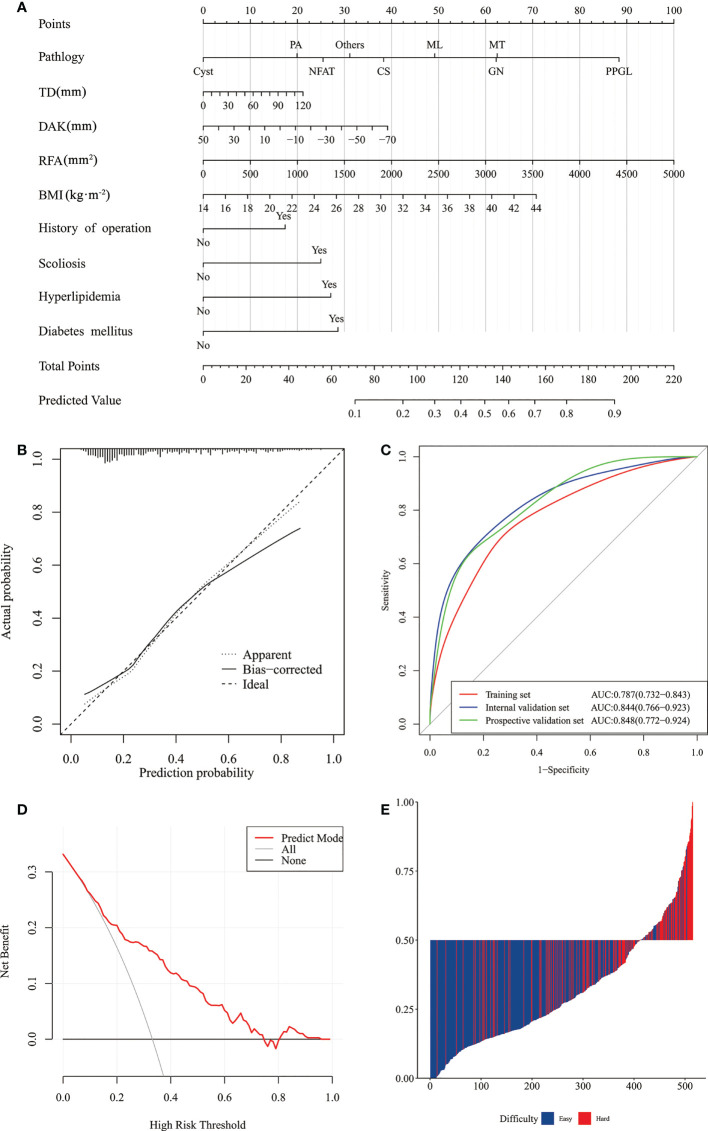
Nomogram of prediction model of difficulty of retroperitoneal laparoscopic adrenalectomy and its performance. **(A)** Nomogram. **(B)** Calibration curves of the nomogram in the training and internal validation sets. **(C)** ROC curves of the nomogram in the training, internal and prospective validation sets. **(D)** Clinical decision curve analysis of prediction model. **(E)** The calculated risk scores for each patient within the combined training and external validation datasets.) (NFAT, non-function adrenal tumor; PA, primary aldosteronism; PPGL, pheochromocytoma and paraganglioma; Others, include eosinophil tumor, teratoma, schwannoma, hematoma, tuberculoma, foreign body granuloma, retroperitoneal bronchial cyst, and hemangioma; MT, Malignant tumor, include adrenocortical carcinoma and adrenal metastasis; TD, tumor diameter; DAK, distance from adrenal tumor to upper pole of kidney; RFA, retrorenal fat area; BMI, body mass index.).

According to the influencing factors screened by Lasso-logistic regression, multivariate logistic regression was carried out, and the results were displayed by nomogram (**Figure 4A**). To use the nomogram, we added up the scores corresponding to each prediction index of the patient to calculate the total score, and determined the corresponding risk value from the total score line, which was the probability that the operation would be more complicated in that patient. For example, if a patient’s pathological type of tumor were PA, with TD of 40 mm, DAK of -10 mm, RFA of 2000 mm^2^, BMI of 26 kg · m^-2^; there was no previous surgery history, scoliosis, history of hyperlipidemia; and there was a history of diabetes, the corresponding scores of each feature were approximately 20, 8, 20, 40, 29, 0, 24, 0, 29, respectively. Thus, the total score was 170, which corresponds to a probability of 80% for increased surgical difficulty and suggests that additional perioperative preparations would be required for an operation.

### Validation and performance of nomogram

The C-index of the model was 0.784 (95% CI: 0.736–0.832). After 1000 resampling internal validations, the visible calibration curve fit the ideal curve well, indicating that the probability of the predicted surgical difficulty of the model had good agreement with the actual situation (**Figure 4B**).

ROC curves were drawn according to the model, with an AUC of 0.787 (95% CI: 0.732–0.843) in the training set and an AUC of 0.844 (95% CI: 0.766–0.923) in the internal validation set. The AUC in the prospective validation set was 0.848 (95% CI: 0.772–0.924) and showed high predictive power for this machine learning model (**Figure 4C**).

The prediction model based on DCA yielded a net benefit for patients of around 15% when the intervention was performed at approximately 35% probability of difficulty (**Figure 4D**). The model had a sensitivity of 0.759, a specificity of 0.720, and a Youden index of 0.479, showing high prediction accuracy (**Figure 4E**).

## Discussion

Adrenal tumors are a hot topic in the field of medicine at present. This study outlined the factors in predicting the difficulty of RPLA *via* nomogram regarding BMI, diabetes mellitus, scoliosis, hyperlipidemia, history of surgery, TD, DAK, RFA and pathology. We concluded that the model had a high predictive ability by internal and prospective validation.

Numerous studies have highlighted that TD is a major factor contributing to the difficulty of RPLA (13, 14, 19–21, 23, 24). In a previous analysis of 275 patients who underwent laparoscopic adrenalectomy, Natkaniec et al. posited that type of pathology was a predictor of difficulty and suggested that surgery for malignancy was much more difficult than that for other tumor types (OR, 3.67, 95% CI 1.52–8.87; P = 0.008) (19), while Vidal (21) and Pisarska (25) considered that surgery was more difficult in pheochromocytoma. The effect of DAK on the difficulty of RPLA was first suggested by Wang (23) (OR, 5.76 95% CI, 2.03–16.35; P = 0.001) and Rah (13) (OR, 3.79; 95% CI, 1.66–8.67; P = 0.002), respectively, and DARP was similarly found to affect surgical difficulty in the former study (OR, 6.23; 95% CI, 2.11–18.38; P = 0.001).

Aside from the tumor itself, the surrounding area and surgical site also have an impact on the difficulty, including adherent perirenal fat (APF) (26), peripheral fat distance (27), and posterior adiposity index (13). Previously, Davidiuk et al. proposed the Mayo adhesive probability (MAP) to predict APF (26), and Kira et al. (24) confirmed MAP to be related to difficulty of RPLA as the adrenal gland is adjacent to the kidney. At the same time, the surrounding environment, fat thickness, and adhesion degree are related. Hence, it is also effective in adrenalectomy. Rah et al. (13) measured periadrenal fat volume and named it as an independent factor affecting difficulty of RPLA. However, the adrenal gland has minimal surrounding fat and significant measurement inaccuracy. Therefore, we synthesized previous studies and concluded that RFA has a considerable impact on the difficulty of RPLA. Patients with diabetes, hyperlipidemia or previous surgery are often more likely to have APF, and thus present with more difficulty in an RPLA procedure (28). As another factor, while BMI is used to evaluate degree of obesity, it primarily reflects body fat composition, and the distribution of visceral fat, especially perirenal fat, may differ. Therefore, the prediction of BMI on difficulty of RPLA is still controversial, with a limited number of studies suggesting that BMI has a significant impact (1, 14, 21).

For patients with scoliosis, the surgical area is smaller which restricted the movement of laparoscopic instruments during the operation. The intercostal space is narrow while the tumor is adjacent to major blood vessels such as the abdominal aorta and splenic veins, which increase the surgical difficulty and make the operation challenging. However, no current relevant cohort or case-control study has been published, and this issue has only been acknowledged in some medical records or case reports (29).

Based on clinical data and CT features, this study developed a prediction model of the difficulty of RPLA by LASSO. It carried out relevant internal and prospective validation, proving that this model can improve the net benefit rate of patients by up to 15%.

At present, some scholars have worked on the prediction of the surgical difficulty of RPLA, but a lack of precise prediction models has been published. Thus, the innovation of this study is that the influencing factors of difficulty were analyzed by LASSO regression, and a prediction model was established and internally and prospectively validated. Further, this study is currently the largest cohort regarding the prediction of the difficulty of RPLA.

Limitations of this study: 1) this study set up an internal validation set and a prospective validation set, which requires further validation in future multicenter studies, 2) LASSO was used in this study, while other machine learning algorithms such as xGBoost and SVM will be used in the further research.

## Conclusion

In this study, independent influencing factors for the difficulty of RPLA included BMI, diabetes mellitus, scoliosis, hyperlipidemia, history of surgery, TD, DAK, RFA, and pathology. Based on the clinical and radiological characteristics, the machine learning prediction model of the difficulty of RPLA established by LASSO regression had a good predictive performance. Using this model can effectively assist surgeons in evaluating the difficulty of RPLA, facilitating complete individualization of perioperative preparation, thereby reducing surgical risk and benefiting patients.

## Data availability statement

The raw data supporting the conclusions of this article will be made available by the authors, without undue reservation.

## Author contributions

SS: Project development, Data collection, Data management, Data analysis, Manuscript writing. JW: Project development, Manuscript writing. BY: Project development, Manuscript writing. YW: Data collection, Data management, Data analysis, Manuscript writing. WY, PY, XN, AF, and LZ: Data collection, Data management. LY and WC: Manuscript editing. YZ: Project development, Data analysis, Manuscript editing, Result inspection. All authors contributed to the article and approved the submitted version.

## Conflict of interest

The authors declare that the research was conducted in the absence of any commercial or financial relationships that could be construed as a potential conflict of interest.

## Publisher’s note

All claims expressed in this article are solely those of the authors and do not necessarily represent those of their affiliated organizations, or those of the publisher, the editors and the reviewers. Any product that may be evaluated in this article, or claim that may be made by its manufacturer, is not guaranteed or endorsed by the publisher.

## Abbreviations

APF, adherent perirenal fat; AUC, area under the curve; BMI, body mass index; CI, confidence interval; CT, computed tomography; DAK, distance from adrenal tumor to upper pole of kidney; DARP, distance from adrenal tumor to renal pedicle; DCA, decision curve analysis; LASSO, least absolute shrinkage and selection operator; MAP, Mayo adhesive probability; OR, odds ratio; PA, primary aldosteronism; POHS, postoperative hospital stay; PPGL, pheochromocytoma and paraganglioma; RFA, retrorenal fat area; ROC, receiver operating characteristics; RPLA, retroperitoneal laparoscopic adrenalectomy; TD, tumor diameter; TPLA, transperitoneal laparoscopic adrenalectomy.
